# "Do my qPCR calculation", a web tool

**DOI:** 10.6026/97320630015369

**Published:** 2019-05-15

**Authors:** Jeremy Tournayre, Matthieu Reichstadt, Laurent Parry, Pierre Fafournoux, Celine Jousse

**Affiliations:** 1 Universite Clermont Auvergne, INRA, UNH, Unite de Nutrition Humaine, CRNH Auvergne, F-63000 Clermont-Ferrand, France; 2 Universite Clermont Auvergne, INRA, VetAgro Sup, UMR1213 Herbivores, Unite Mixte de Recherche sur les Herbivores, F-63000 Clermont-Ferrand, France

**Keywords:** qPCR, automation tool, web server

## Abstract

In order to automatically process qPCR raw data, we present the tool "Do my qPCR calculation". We offer a website to automatically calculate the data normalization and represent the different samples graphically in an Excel file. This tool is also available on Github for installation and local use with or without web interface.

## Background

The fluorescence-based quantitative real-time PCR (qPCR) is a molecular biology technique that is routinely used in laboratories. It quantitatively monitors, in real-time, the amplification of a targeted DNA (or cDNA after reverse transcription of a target RNA) molecule during the PCR. The quantification is based on the number of cycles of PCR at which the fluorescence exceeds a given threshold (Cq). Softwares associated with qPCR apparatus provides Cq for each sample and usually propose a tool for calculation for quantification and normalization for each sample. However, the use of such specific software often requires a license and/or is restricted to a given operating system. For example, CFX Maestro - Software license from Bio-Rad goes from 150 euros for the Mac version to 1000 euros for the Windows version (catalog price seen on 08/02/2019).

Given these constraints, researchers very often extract Cq from the computer connected to the thermocycler in order to carry out their own calculations in their own spreadsheet (for example Excel, LibreOffice, ...) which is tedious. To go further, researchers have built free tools to perform automatic calculations: for example, QPCR and LinRegPCR. QPCR requires an install on a server and a license request 1. LinRegPCR is an executable running only on Windows 2. Unfortunately, it does not propose an example or template file to understand how the input file should be processed. Finally, to our knowledge, there is no free tool available on any plateform without the need to install a particular device to analyze qPCR data. That's why we present our tool: "Do my qPCR calculations".

"Do my qPCR calculations" can be used through a website hosted on a server that provides computing power. "Do my qPCR calculations" allows, from Cq, to calculate almost instantaneously in an excel file the relative quantities of RNA normalized by a reference gene. It allows taking into account groups of samples to perform student test between the control group and experimental groups. Also the tool makes histograms of each result. Input data will only be used for generate results and not for any further purpose. They are automatically deleted from the server when the Excel file is generated. We use the recommended MIQE nomenclature 3, i.e Cq, reference gene.

## Methodology

"Do my qPCR calculation" is built from Perl with librairies: Excel::Writer::XLSX, Spreadsheet::ParseExcel and Statistics::TTest. The ssconvert tool is used to convert xlsx/xls/ods files into .tsv format.The web interface is a PHP script containing HTML, PHP, CSS and javascript with jQuery, Jexcel and jQuery-csv libraries.

**Figure 1:**
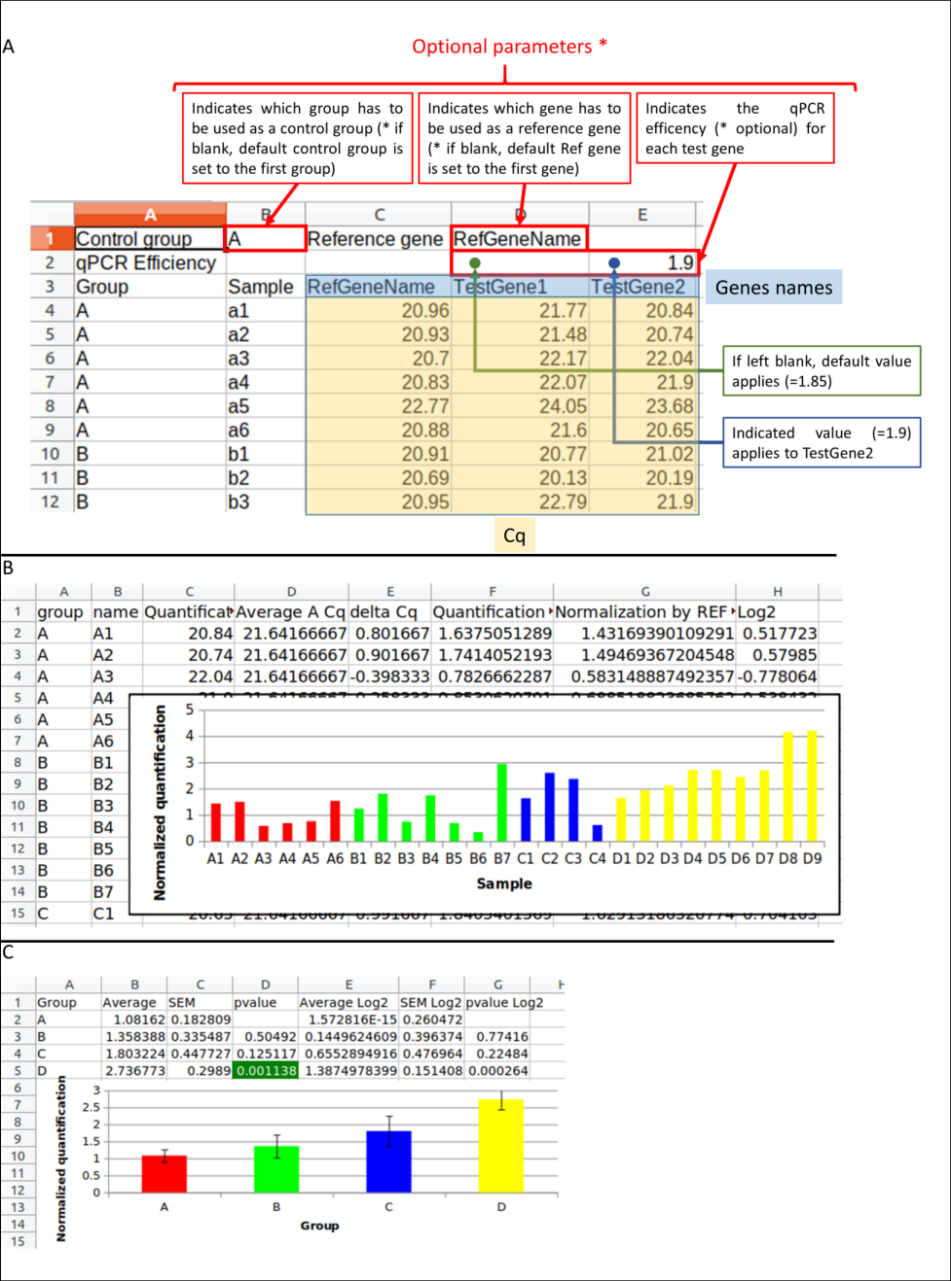
A) The input data has to be in a file in .tsv, .xls, .xlsx. or .odt format or written in the web form. The first two rows contain options. The control group is defined in B1 cell: the "A" group is chosen. The reference gene in D1 cell: the "RefGeneName" gene is selected. The second row allows defining the qPCR efficiency for each gene written on the third row. On the third row there are two column headers: "Group" and "Sample" and then there are the genes: "RefGeneName", "TestGene1", "TestGene2". The other rows correspond to the data table: sample according to the Cq. Submitting this file to "do my qPCR calculation" allows you to obtain the result file in Excel format. B) The Excel file contains the normalized Cq for each sample with histograms. C) The Excel file also contains the average results and the student test for each experimental group between the control groups

Using the tool Figure 1 shows the input and output using an example for the tool

### Input: 

"Template" contains a description of the input file. We offer one example, ready to be submitted, that can be downloaded in tabulated separated value .tsv, Excel or in OpenOffice/LibreOffice format. The first two rows permit to specify parameters such as the control group, the reference gene and the qPCR efficiency for each gene. By default: the first group corresponds to the control group, the first gene corresponds to the reference gene and the efficiency is set to 1.85 (92.5% efficiency). If applicable, the user can indicate a custom efficiency right above the gene name. The data has to be given in column with the following headers: "Group", "Sample" and the different amplified genes. Missing values (other than a number, i.e NA, empty...) may be present in the file; they will not be taken into account into calculations.

### Output: 

The generated excel file contains two tabs for each analyzed gene. One tab contains the analysis done per sample and the next tab contains the average for each group. In the case of the analysis by sample, the table contains: (A) groups, (B) name of each sample, (C) Cq corresponding to the initial data, (D) average Cq of the control samples which permit to calculate (E) the delta Cq calculated as (D) - (C), (F) quantification calculated using the efficiency set in the options (eff) and the delta Cq (E) with the formula: eff^(E), (G) normalized quantification by the reference gene, and (H) the log2 corresponding to the normalized quantification by the reference gene set in log2. The average tab contains: (A) groups, (B) average of the samples of the group, (C) SEM, (D) pvalue of the student test between the experimental group and the control group, (E, F and G) the results calculated with the log2 data. In each analysis histograms are generated to visualize the data.

## Conclusion

Our tool "Do my qPCR calculation" was created to provide to the scientific community a simple and efficient way to deal with qPCR data. In order to make it accessible to a maximum of users the tool is available on a website accessible on any Internet browser commonly used whatever the operating system and type of platform, i.e Windows, Mac... Moreover our tool provides results as a spreadsheet that can be dealt with any spreadsheet software. This tool is meant to be further developed if needed by taking into account the comments of users while keeping the tool easy to use.

## Availability

The data is available at http://147.99.156.182/do_my_qPCRcalc and the source code is made available at https://github.com/JeremyTournayre/do_my_qPCRcalc
